# Lack of an apparent role for endothelin‐1 in the prolonged reduction in renal perfusion following severe unilateral ischemia‐reperfusion injury in the mouse

**DOI:** 10.14814/phy2.13027

**Published:** 2016-11-15

**Authors:** Erika I. Boesen

**Affiliations:** ^1^ Department of Cellular and Integrative Physiology University of Nebraska Medical Center Omaha Nebraska

**Keywords:** Endothelin‐1, ischemia, kidney, renal perfusion, ultrasound

## Abstract

Therapeutic approaches to block the progression from acute kidney injury to chronic kidney disease are currently lacking. Endothelin‐1 (ET‐1) is a powerful vasoconstrictor, induced by hypoxia, and previously implicated in renal ischemia‐reperfusion (IR) injury. This study tested the hypothesis that blunting the vascular influence of ET‐1, either through endothelin ET_A_ receptor blockade (ABT‐627) or vascular endothelial cell deletion of ET‐1 (VEET KO), would improve recovery of renal perfusion and repair of injury following a severe ischemic insult in mice (45 min unilateral renal ischemia). Male C57Bl/6 mice receiving vehicle or ABT‐627 commencing 2 days prior to surgery, and VEET KO mice and wild‐type littermates (WT) underwent 45 min unilateral renal IR surgery followed by 28 days recovery. Renal blood velocity was measured by pulsed‐wave Doppler ultrasound before and after surgery. Renal blood velocity was not significantly different between pairs of groups before surgery. Unilateral IR induced a marked reduction in renal blood velocity of the IR kidney at 24 h postsurgery in all groups, which partially recovered but remained below baseline at 28 days post‐IR. Despite the lack of effect on renal blood velocity, ET_A_ receptor blockade significantly attenuated the atrophy of the post‐IR kidney, whereas this was not significantly affected by lack of endothelial ET‐1 expression. These data suggest that although blockade of the ET_A_ receptor is mildly beneficial in preserving renal mass following a severe ischemic insult, this protective effect does not appear to involve improved recovery of renal perfusion.

## Introduction

Acute kidney injury (AKI) is of grave clinical concern, with a mortality rate of ~50% (Mehta 2003) and no effective treatments currently available. Furthermore, there is growing evidence that patients who do survive an episode of AKI carry an increased risk of later developing chronic kidney disease (CKD), with a recent meta‐analysis (Coca et al. 2012) showing a graded increase in risk of CKD with severity of AKI, and estimating the pooled average risk to be approximately eightfold. Given the substantial morbidity and mortality associated with CKD, poor quality of life enjoyed by patients who progress to end‐stage renal disease (ESRD), as well as the cost of care associated with both CKD and ESRD, a greater understanding of the mechanisms underlying progression from AKI to CKD and identification of possible therapeutic targets are needed.

In comparison to the large body of research on AKI per se, which frequently focuses on the first 24–48 h following injury, AKI to CKD progression remains a vastly understudied area but is gaining interest (e.g., as discussed in (Basile et al. 2016; Dhaun and Webb 2013; Venkatachalam et al. 2015)). Inadequate perfusion of the kidneys, for example, due to surgical procedures or trauma, is a common cause of AKI in the developed world (Liano and Pascual 1996; Mehta et al. 2004). Studies of animal models utilizing unilateral renal ischemia and reperfusion (IR) with the contralateral kidney left intact have begun to shed some light on the possible pathogenic mechanisms involved in the progression from AKI to CKD, since the presence of the functioning contralateral kidney avoids mortality due to AKI, but the IR kidney goes on to lose renal mass and becomes fibrotic (Le Clef et al. 2016). One promising candidate for involvement in AKI to CKD progression identified so far is endothelin‐1 (ET‐1), which is produced by multiple cell types in the kidney, including endothelial and tubular epithelial cells (Kohan 1996). ET‐1 acts in a paracrine/autocrine manner rather than as a hormone, via ET_A_ and ET_B_ receptors, which have distinct cell‐specific effects (Kohan et al. 2011). ET_A_ and ET_B_ receptors on vascular smooth muscle mediate powerful vasoconstriction (Just et al. 2004). ET_B_ receptors on vascular endothelium and tubular epithelium stimulate NO and prostaglandin release, inducing vasodilation and inhibiting salt and water reabsorption, respectively (Kohan et al. 2011). In addition to these physiological actions of ET‐1, when ET‐1 is overexpressed in the kidney pathophysiological results include glomerulosclerosis and renal fibrosis (Hocher et al. 1997). Renal IR injury involves oxidative stress and provokes an inflammatory response, and limited studies suggest that these may be partially mediated by ET‐1 (Forbes et al. 1999; Erdogan et al. 2006; Gulmen et al. 2009; Arfian et al. 2012). The results of a recent study of unilateral renal IR in a mouse model suggest that ET‐1 and ET_A_ receptors in particular play an important role in promoting the transition from AKI to CKD (Zager et al. 2013), however, the precise mechanisms involved remain to be determined.

While reperfusion following renal ischemia is itself associated with a burst of oxidative stress, increasing lipid peroxidation (Paller et al. 1984), longer‐term following IR the postischemic kidney displays prolonged vasoconstriction, which is thought to exacerbate hypoxia (Schrier et al. 2004), and in turn may contribute to progression of renal injury. As a powerful, long‐lasting vasoconstrictor (Yanagisawa et al. 1988) that is upregulated in the kidney by ischemia (Wilhelm et al. 1999; Forbes et al. 2001b) ET‐1 is a strong candidate for impairing recovery of renal perfusion following IR. The contribution of ET‐1 to long‐term changes in renal hemodynamics following IR injury has not, however, been addressed. IR increases immunoreactive ET‐1 in the endothelium post‐IR (Wilhelm et al. 1999), and the abluminal nature of ET‐1 release from endothelial cells (Wagner et al. 1992) ideally positions endothelial‐derived ET‐1 to promote vasoconstriction following IR. Moreover, marked and prolonged increases in both ET‐1 and ET_A_ receptor expression in the kidney following unilateral renal IR have been reported (Zager et al. 2013), although which cell types are involved is not known. The aim of this study was therefore to test whether blockade of ET_A_ receptors or endothelial cell‐specific deletion of ET‐1 improves long‐term recovery of renal perfusion following IR.

## Methods

Experiments were conducted on male C57Bl/6 mice obtained from Jackson Laboratories, Bar Harbor, ME at Georgia Health Sciences University (now Augusta University), Augusta, GA, and on male endothelial cell‐specific ET‐1 knockout mice (VEET KO; ET‐1^flox/flox^ and Tie2‐Cre^+^) and wild‐type littermates (WT; ET‐1^flox/flox^ and Tie2‐Cre^−^) bred and genotyped as described previously (Kisanuki et al. 2001) at the University of Nebraska Medical Center, Omaha, NE from ET‐1^flox/flox^ founders obtained from Dr. Donald Kohan, University of Utah, and Tie2‐Cre mice from Jackson Laboratories, both on a C57Bl/6 background. The procedures were approved in advance by the Animal Care and Use Committees of the respective institutions. Mice were housed individually and maintained on a normal salt diet (0.3% sodium; Harlan Teklad, Madison, WI) throughout the experiment. For studies of long‐term outcomes of unilateral renal IR, one group of C57Bl/6 mice received ABT‐627 (“atrasentan”; Abbvie Inc., Abbott Park, IL) in their drinking water (10 mg/kg/d p.o.) throughout the experiment, commencing 2 days prior to baseline measurements of renal perfusion (as described below). This strategy of commencing ABT‐627 administration prior to IR surgery was adopted to ensure blockade of ET_A_ receptors at the time of injury since ET‐1 is rapidly upregulated by ischemia (observable within 1 h of reperfusion, as discussed below). All other mice received normal drinking water.

In an initial experiment, the effect of 45 min unilateral renal IR on renal vascular ET‐1 expression was determined. Male C57Bl/6 mice underwent unilateral renal IR surgery following procedures similar to described previously (Boesen et al. 2012). Mice were anesthetized with isoflurane (~2% by inhalation) and placed in a prone position on a servo‐controlled heating table to maintain core body temperature at 37°C. The right kidney was approached by a retroperitoneal incision and a small microvascular clamp was applied to the renal artery for 45 min, stopping blood flow. The clamp was then removed, allowing reperfusion of the kidney for 1 h, after which mice were sacrificed and renal vascular tissue was collected by gentle sieving through a 100 *μ*m mesh, and RNA extracted using an RNeasy Mini kit (Qiagen, Valencia, CA) and reverse transcribed to cDNA using a QuantiTect Reverse Transcription kit. Renal vascular expression of ET‐1 mRNA relative to GAPDH was assessed using QuantiTect Primer assays and relative‐fold expression was calculated as 2^−ΔΔCT^ as described previously (Boesen et al. 2012).

For studies of long‐term outcomes of unilateral renal IR, mice underwent ultrasound imaging 2–3 days prior to unilateral renal IR surgery (see below) to obtain a baseline measurement of renal perfusion, and again following IR surgery at 24 h post‐IR and at several time points over a 28‐day recovery period (3, 7, 14, and 28 days post‐IR) to provide time‐course information. Ultrasound imaging was performed as previously described (Boesen et al. 2012). Mice were anesthetized with isoflurane (~1.5% by inhalation; Baxter, Deerfield, IL) and placed in a supine position on the THM100 MousePad imaging platform (Indus Instruments, Houston, TX) and body temperature maintained at 37°C. Depilatory cream (Nair TM, Carter‐Horner, Mississauga, Canada) was used to remove fur from the abdominal skin and medical ultrasound acoustic gel (Other‐Sonic, Pharmaceutical Innovations, Inc., Newark, NJ) was used as a coupling fluid between the real‐time microvisualization (RMV) scanhead and the skin. Ultrasound imaging was performed using the Vevo 770 system (VisualSonics Inc., Toronto, Canada) with an RMV‐706 scanhead for C57Bl/6 mice or RMV‐704 scanhead for VEET KO and WT mice (both 40 MHz, 6 mm focal length), which was positioned and held immobile using the VisualSonics Vevo Integrated Rail System. Peak systolic renal artery blood velocity, end diastolic blood velocity, and mean blood velocity (calculated from the velocity–time integral divided by time) for each kidney were measured by pulsed‐wave Doppler (PW mode) for two periods of 5–10 cardiac cycles with values averaged from each.

All mice underwent unilateral renal IR surgery following procedures similar to described previously (Boesen et al. 2012). Mice were anesthetized with isoflurane (~2% by inhalation) and placed in a prone position on a servo‐controlled heating table to maintain core body temperature at 37°C. The right kidney was approached by a retroperitoneal incision and a small microvascular clamp was applied to the renal artery for 45 min, stopping blood flow. The clamp was then removed, allowing reperfusion of the kidney, and 0.4 mL sterile saline was instilled i.p. to replace fluids. The retroperitoneal incision was closed using sterile sutures and surgical staples. Anesthesia was withdrawn and the mouse was allowed to recover, with analgesia provided by buprenorphine (0.1 mg/kg s.c.; Reckitt Benckiser Healthcare (UK) Ltd., Hull, England).

Following the final ultrasound measurement at 28 days, mice were deeply anesthetized (pentobarbital sodium at 60 mg/kg, Abbott, North Chicago, IL or ~5% isoflurane by inhalation), the mice exsanguinated and their kidneys collected for analysis. After recording their weights, kidneys were immersion fixed for 24 h in 10% neutral buffered formalin, then paraffin embedded and sectioned for histological staining (Masson's Trichrome). Eighteen to twenty nonoverlapping images of renal cortex and five to ten images of outer medulla (except in one mouse where only three images were possible) were captured using 20× objective lens and a Digital Sight DS‐5M‐L1 system (Nikon, Melville, NY) fitted to an Axioskop 20 light microscope (Carl Zeiss, Thornwood, NY); brightness and contrast were adjusted using Microsoft PowerPoint 2010, with all images treated identically. Images were evaluated for percentage areas occupied by viable tubules (counted by overlaying a 10 × 10 grid of points onto the image) and fibrosis (color thresholding) using ImageJ software.

Statistical analysis was performed using GraphPad Prism 6. Renal vascular ET‐1 mRNA expression was compared between IR and contralateral kidneys by paired Student's *t*‐test. Comparison of contralateral:IR kidney weight ratios between groups was by unpaired Student's *t*‐test. Renal blood velocity measurements in either the contralateral or IR kidneys were compared by two‐factor repeated‐measures analysis of variance (ANOVA), testing for main effects of group (*P*_Group_), time (*P*_Time_), and the interaction between the two (*P*_G*T_). Histological scoring data and kidney‐to‐bodyweight ratios were compared by two‐factor ANOVA, testing for main effects of group (*P*_Group_), kidney (i.e., contralateral vs. ischemic; *P*_Kidney_), and the interaction between the two (*P*_G*K_). Raw kidney and bodyweights were compared between groups by unpaired Student's *t*‐test. Data are presented as mean ± SEM, with *P* < 0.05 considered statistically significant.

## Results

**Figure 1:**
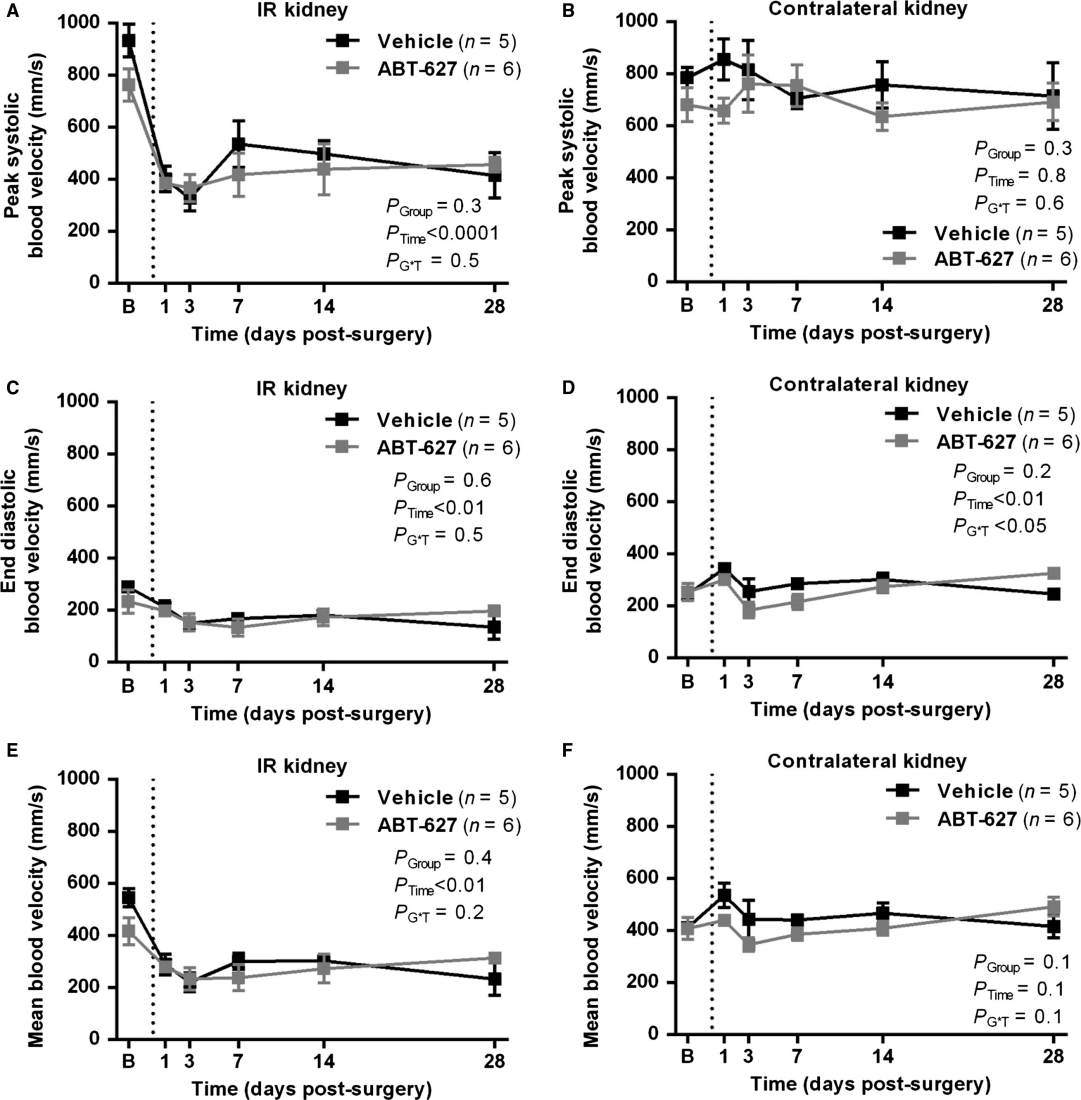
Time course of changes in renal perfusion in vehicle and ABT‐627‐treated C57Bl/6 mice undergoing the unilateral renal IR model. Data presented were obtained by noninvasive pulsed‐wave Doppler ultrasound, measured prior to (“B”) and following unilateral renal IR surgery (time of surgery indicated by dashed line). Data shown are mean ± SEM for peak systolic velocity (A and B), end diastolic velocity (C and D), and mean blood velocity (E and F) in the IR and contralateral kidneys as indicated. *P* values were determined by two‐factor repeated‐measures ANOVA, testing for main effects of group (*P*_Group_), time (*P*_Time_), and whether the response over time differed between groups (*P*_G*T_). IR, ischemia reperfusion.

We had previously observed using ultrasound that, following 1 h unilateral ischemia in mice, renal peak systolic blood velocity remains markedly suppressed at 1 h and 24 h of reperfusion (Boesen et al. 2012). In this study, a shorter ischemic period of 45 min was used, which is in the midrange of ischemic times used in the unilateral IR model in mice (Le Clef et al. 2016). An initial experiment was performed in C57Bl/6 mice to determine whether this stimulus produces an increase in ET‐1 expression. Following 45 min unilateral ischemia and 1 h reperfusion, renal vascular ET‐1 mRNA was found to be significantly increased in the IR kidney compared to the contralateral kidney (2^−ΔΔCT^ of 2.6 ± 0.4 compared to 1.2 ± 0.3, *n* = 8, *P* < 0.05). In subsequent experiments, the long‐term consequences of 45 min unilateral IR were studied. As shown in Figure 1A, unilateral IR induced a marked reduction in renal peak systolic blood velocity at 24 h post‐IR in both vehicle and ABT‐627‐treated groups (44 ± 6 and 52 ± 5% of baseline, respectively). Recovery of perfusion was still poor at 28 d postsurgery (47 ± 11 and 61 ± 4% of baseline). No significant change in renal peak systolic blood velocity was observed over time in the contralateral kidney, or was there any significant difference between vehicle‐ and ABT‐627‐treated groups (Fig. 1B). Effects on end diastolic blood velocity (Fig. 1C and D) and mean blood velocity (Fig. 1E and F) followed a largely similar pattern, with marked reductions in velocity in the IR kidney following ischemia and incomplete recovery (*P*_Time_ < 0.01), with no significant difference between groups (*P*_Group_ > 0.05), and little change in the contralateral kidney except for some fluctuation over time which reached statistical significance for end diastolic velocity (Fig. 1D).

**Figure 2:**
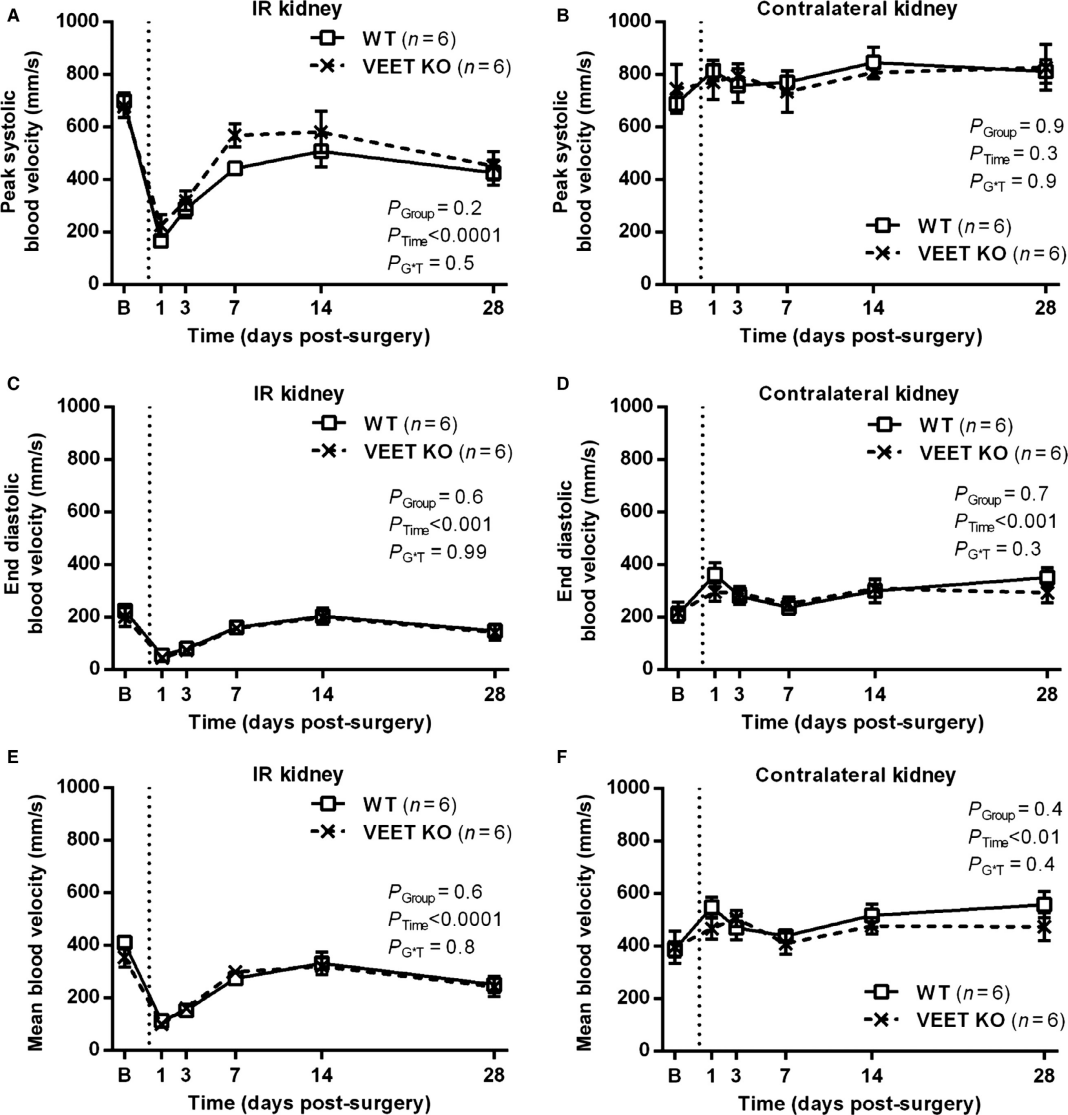
Time course of changes in renal perfusion in WT and VEET KO mice undergoing the unilateral renal IR model. Data presented were obtained by noninvasive pulsed‐wave Doppler ultrasound, measured prior to (“B”) and following unilateral renal IR surgery (time of surgery indicated by dashed vertical line). Data shown are mean ± SEM for peak systolic velocity (A and B), end diastolic velocity (C and D), and mean blood velocity (E and F) in the IR and contralateral kidneys as indicated. *P* values were determined by two‐factor repeated‐measures ANOVA, testing for main effects of group (*P*_Group_), time (*P*_Time_), and whether the response over time differed between groups (*P*_G*T_). IR, ischemia reperfusion.

A similar pattern of results was observed in WT and VEET KO mice, with a marked reduction in peak systolic blood velocity observed in the IR kidney at 24 h post‐IR in both groups, and despite a partial rebound toward baseline levels recovery remained incomplete even after 28 days (61 ± 6 and 69 ± 12% of baseline, respectively; Fig. 2A). No significant change was observed in peak systolic velocity in the contralateral kidney over time or between groups (Fig. 2B). End diastolic velocity was also reduced following IR (*P*_Time_ < 0.001) and although it too appeared to rebound, it remained depressed at 28 days (69 ± 11 and 76 ± 18% of baseline in WT and VEET KO mice, respectively; Fig. 2C). There was a gradual increase in end diastolic velocity in the contralateral kidneys of both groups over time (*P*_Time_ < 0.001), but this effect was not significantly different between groups (Fig. 2D). Similar results were observed for mean blood velocity with incomplete recovery observed in the IR kidney (*P*_Time_ < 0.0001; Fig. 2E) and a modest increase over time in the contralateral kidney (*P*_Time_ < 0.01; Fig. 2F), but no significant difference between groups.

**Figure 3:**
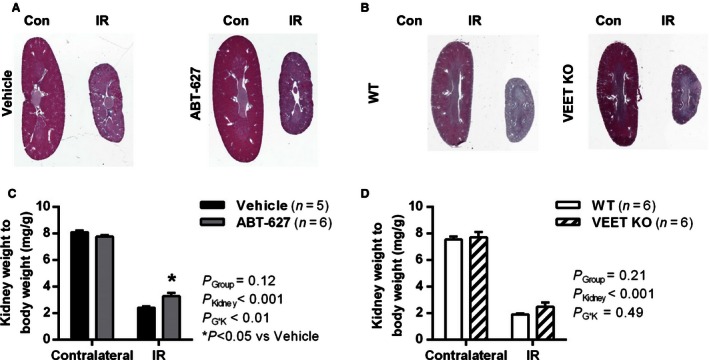
Kidney size at 28 days postsurgery. Representative low‐magnification images of Masson's Trichrome‐stained sagittal sections of IR‐injured kidneys (IR) and untouched contralateral kidneys (Con) are shown for (A) vehicle‐ or ABT‐627‐treated C57Bl/6 mice and (B) VEET KO and WT mice, with corresponding kidney weight‐to‐bodyweight ratios summarized in (C) and (D). Data shown are mean ± SEM. *P* values were determined by two‐factor ANOVA, testing for main effects of group (*P*_Group_), kidney (*P*_Kidney_), and the interaction between the two (*P*_G*K_). *P < 0.05 compared with vehicle IR kidney. IR, ischemia reperfusion.

At 28 days recovery post‐IR surgery, there was a significant change in the size of the ischemic versus contralateral kidneys of both groups, with IR kidneys of all groups undergoing marked atrophy, as evident from low‐power images of kidney sections (Fig. 3A and B) and kidney weight‐to‐bodyweight ratios (Fig. 3C and D). The same patterns of results were seen when raw kidney weights were analyzed, and there were no significant differences in bodyweights of vehicle compared to ABT‐627‐treated mice, or of WT compared to VEET KO mice at day 28 (Table 1). IR kidney weight was better preserved in ABT‐627‐treated mice compared to vehicle‐treated mice (*P* < 0.05; Table 1). Consequently, the difference in kidney weights between IR and contralateral kidneys was significantly attenuated in ABT‐627‐treated mice (*P*_G*K_ < 0.01; Fig. 3C), which displayed an IR:Contralateral kidney weight ratio of 0.42 ± 0.03 compared to 0.30 ± 0.01 in vehicle‐treated mice (*P* = 0.01). VEET KO mice did not display any significant attenuation of the change in kidney weights between contralateral and ischemic kidneys (*P*_G*K_ = 0.49; Fig. 3D), with raw kidney weights not significantly different between groups (Table 1) and an IR:Contralateral kidney weight ratio of 0.32 ± 0.03 compared to 0.25 ± 0.01 in WT mice (*P* = 0.1).

**Table 1:** Body and kidney weights

Group	Bodyweight (g)	Contralateral kidney weight (mg)	IR kidney weight (mg)
Vehicle (*n* = 5)	26.3 ± 0.6	213 ± 5	64 ± 3
ABT‐627 (*n* = 6)	27.2 ± 0.5	211 ± 4	89 ± 6^a^
WT (*n* = 6)	24.9 ± 0.9	188 ± 8	48 ± 3
VEET KO (*n* = 6)	26.0 ± 0.9	200 ± 10	64 ± 9

a *P* < 0.05 compared to Vehicle group. WT, wild type.

**Figure 4:**
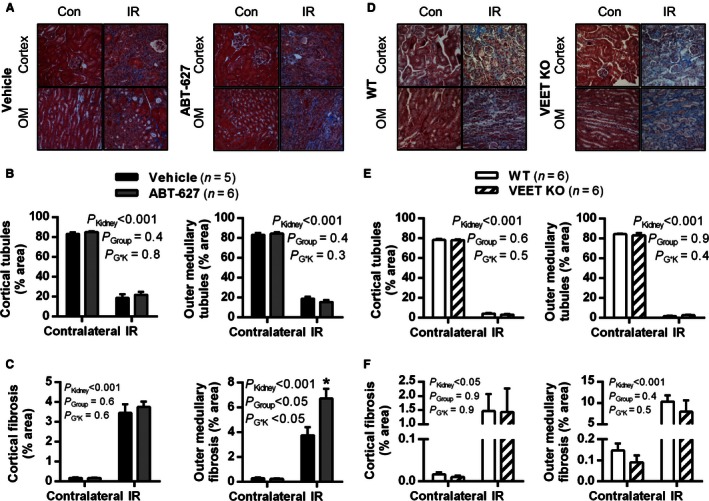
Histological analysis of kidneys at 28 days postsurgery. Top panels (A and D) show representative images of Masson's Trichrome‐stained sections (20× magnification) of IR and contralateral (Con) kidneys of vehicle‐ or ABT‐627‐treated C57Bl/6 mice, and of VEET KO and WT mice. Images from cortex and outer medulla were analyzed for percentage of area occupied by viable tubules (B and E) and for fibrosis (C and F). Data shown are mean ± SEM. *P* values were determined by two‐factor ANOVA, testing for main effects of group (*P*_Group_), kidney (*P*_Kidney_), and the interaction between the two (*P*_G*K_). *P < 0.05 compared with vehicle IR kidney. IR, ischemia reperfusion.

Analysis of Masson's trichrome‐stained kidney sections collected at day 28 revealed marked parenchymal injury to the IR kidneys (Fig. 4A and D), including a loss of renal tubules and increase in fibrosis, in both the renal cortex and outer medulla (*P*_Kidney_ < 0.001; Fig. 4B‐F). These effects were not significantly attenuated by ABT‐627 treatment of C57BL/6 mice, or of deletion of ET‐1 from vascular endothelium. Unexpectedly, outer medullary fibrosis was significantly worsened in the IR kidneys of ABT‐627‐treated mice (*P* < 0.05; Fig. 4C).

## Discussion

In this study, it was observed that 45 min unilateral renal IR resulted in rapid upregulation of renal vascular ET‐1, persistent reductions in renal perfusion postischemia, and marked atrophy of the kidney, including loss of tubules and fibrosis as assessed 28 days later. Pharmacological blockade of the ET_A_ receptor or deletion of ET‐1 from the endothelium had no significant effect on restoration of renal perfusion and little (ET_A_ receptor blockade) to no (VEET KO) significant effect on preservation and long‐term recovery of the injured kidney. Accordingly, targeting the endothelin system appears insufficient to blunt the progression of renal injury following a moderately severe ischemic insult.

Interestingly, the current results, obtained in mice on the C57BL/6 background after a 4‐week follow‐up period, contrast with the earlier work of Zager and colleagues (Zager et al. 2013) wherein CD‐1 mice that underwent 30 min unilateral renal ischemia showed well‐preserved renal mass when treated with ABT‐627, as assessed 2 weeks following injury. It could be argued that the model used in this study may be too severe to be responsive to therapy. Interestingly, while a partial recovery of renal perfusion was evident in WT and VEET KO mice at 14 days, perfusion appeared to be going into decline again by day 28 (Fig. 2). In addition to ischemic time, several other differences in the experimental design of the two studies may have contributed to the differing results, including follow‐up time, operative factors such as anesthetic choice or temperature control, and mouse strain with C57Bl/6 being more susceptible than certain others (Wei and Dong 2012). Several other studies in both rat and mouse models of ischemic renal injury have shown beneficial effects of blocking the ET_A_ receptor, both ET_A_ and ET_B_ receptors, or administration of anti‐ET‐1 antibodies, however, the majority of these studies were in models of AKI and typically with short follow‐up periods (hours to 7 days) (Kon et al. 1989; Shibouta et al. 1990; Mino et al. 1992; Espinosa et al. 1996; Forbes et al. 1999; Kuro et al. 2000; Knoll et al. 2001; Wilhelm et al. 2001; Nishida et al. 2002; Erdogan et al. 2006; Gulmen et al. 2009; Arfian et al. 2012), with a small number continuing to 14 days post‐injury (Gellai et al. 1994; Birck et al. 1998; Braun et al. 2000; Zager et al. 2013), hampering the assessment of the contribution of ET‐1 to long‐term recovery or progression to CKD. It remains possible that ET‐1 plays a greater role in a uremic setting, as induced by bilateral ischemia or unilateral ischemia with contralateral nephrectomy, rather than in the unilateral ischemic model with an intact contralateral kidney compensating for the impaired function of the injured kidney.

With regard to long‐term effects of endothelin receptor blockade, a unique study was performed in female rats that underwent unilateral nephrectomy followed by IR injury to the remaining kidney and then received a one‐time treatment with an ET_A_ receptor‐selective antagonist at 24 h post‐IR, with injury assessed 6 months later (Forbes et al. 2001a). ET_A_ receptor blockade for this brief period had a beneficial effect on fibrosis. In this study no benefit of ET_A_ receptor blockade or even a slight worsening of renal fibrosis in the outer medulla was detected. This finding was unexpected; intrarenal hemodynamics were not assessed and so it is not known whether medullary blood flow was increased and perhaps pressure transmission to the medulla deleteriously enhanced by ABT‐627, although such effects have not been reported to the author's knowledge in hypertensive models, and the ET_A_ receptor has generally been shown to promote rather than inhibit fibrosis development (Kohan et al. 2011). Alternately, it could be argued that an enhanced restoration of renal perfusion immediately following ischemia might enhance injury, however, for practical reasons renal perfusion was not measured immediately following ischemia in this study. Whether it therefore would have been more beneficial to commence ET_A_ receptor blockade after IR surgery rather than before remains uncertain. Direct comparisons of pre‐ versus postinjury timing of endothelin receptor antagonist treatments are rare in the literature, but in the 30 min unilateral ischemia study performed by Zager and colleagues (Zager et al. 2013) pre‐ and postischemic treatment yielded a similar protective effect to just postischemic treatment. It should be noted that in the study by Forbes et al. (Forbes et al. 2001a), the kidneys of both treated and untreated rats had largely recovered by 6 months, whereas in this study the kidneys had progressed to an end‐stage kidney disease‐like morphology. It is possible that with a sufficiently severe insult to the kidney, damage to the kidneys may be too great to be rescued by blocking ET‐1‐mediated actions alone. Given the limited and disparate findings of our study and the small number of previous studies of whether ET_A_ receptor blockade is beneficial in preventing progression to chronic kidney disease following ischemic injury, further investigation of this important therapeutic question is warranted.

The role of the ET_B_ receptor in the long‐term outcome of renal IR injury is poorly understood and was not addressed in this study. Since ET_B_ receptors serve as clearance receptors for ET‐1, and can promote NO release, vasodilation, and inhibit tubular consumption of oxygen (Kohan et al. 2011), one might speculate that ET_B_ receptors would be protective in the setting of renal IR injury. Consistent with this, uninephrectomized ET_B_ receptor‐deficient rats displayed an impaired recovery of renal function post‐IR compared with wild‐type rats (Nishida et al. 2002). Forbes et al. (2001a) found that a once‐only administration of a dual ET_A_ and ET_B_ receptor blocker in rats at 24 h post‐IR had a detrimental effect on GFR, proteinuria, and renal histology 6 months later, whereas ET_A_ blockade alone did not. Recently, the ET_B_ receptor was implicated in mediating the protective effects of mineralocorticoid receptor antagonism at 24 h following bilateral renal IR in rats (Barrera‐Chimal et al. 2016). Together, these studies suggest an important role for the ET_B_ receptor in a critical early‐window post‐IR. In contrast, ET_B_ receptor blockade using BQ‐788 did not worsen the renal atrophy observed by Zager and colleagues (Zager et al. 2013) 2 weeks following unilateral renal IR in CD‐1 mice, although BQ‐788 is a peptide and was delivered via drinking water, likely compromising its biological activity. Thus, the ET_B_ receptor may play a protective role in the response to renal IR injury, but the precise mechanisms involved remain to be elucidated.

An additional goal of these studies was to determine the contribution of endothelial cell‐derived ET‐1 to renal IR injury. Previously, Arfian et al. (2012) reported at 24 h following bilateral renal IR, VEET KO mice displayed an attenuated rise in serum creatinine and tubular injury, oxidative stress, and inflammation. This study did not test whether the initial response 24 h after unilateral renal IR injury was different in WT and VEET KO mice, however, it is interesting to note that there was a trend toward a slightly greater rebound of peak systolic velocity but not end diastolic or mean velocity at 7 days post‐IR in VEET KO compared to WT mice (Fig. 2). We did not, however, detect any reduction in the severity of injury in VEET KO mice at later time points in this model, which as alluded to above, is quite different to the bilateral model, which in rodents typically results in recovery of renal function if the initial period of AKI is survived. Unfortunately our mouse colony produces VEET KO mice at a slow rate, making analysis of tissue at earlier time points or studies of shorter ischemic times impractical. Accordingly, whether a beneficial effect of VEET KO would be seen at an earlier stage of injury or unmasked by a less severe insult remains unknown. A caveat to these studies is that although upregulation of immunoreactive ET‐1 in the endothelium following IR injury has previously been demonstrated (Wilhelm et al. 1999), it should be noted that many other cell types within the kidney also produce ET‐1 (Kohan et al. 2011) and so nonendothelial sources of ET‐1 could also affect the vasculature.

Although the therapeutic approaches tested in this study were unsuccessful in ameliorating renal injury, the results suggest that noninvasive measurements of renal perfusion via ultrasound could be used to provide insight into whether kidneys are undergoing recovery from ischemic insults or are instead progressing to a CKD‐like state. We have previously found that in terms of the percentage reduction in renal perfusion measured 1 h following 1 h ischemia, the relative changes in peak systolic blood velocity measured by noninvasive ultrasound (Boesen et al. 2012) are comparable to those of renal blood flow measured conventionally by perivascular flow probes (Schneider et al. 2010). Visualization of the renal artery wall was not possible with the equipment used in this study, precluding calculations of renal blood flow. It should therefore be noted that these measurements of velocity may provide an index of renal perfusion but are not in themselves measures of volumetric blood flow. Another ultrasound‐derived measurement, resistive index, when taken within 12 h of admission to the intensive care unit, was found to predict development of AKI 3 days later in patients with sepsis or polytrauma (Schnell et al. 2012). However, this measurement might be influenced by fibrotic changes within the renal parenchyma, and so interpretation of changes in this measurement over time may be complex. Whether deterioration in renal perfusion during the development of CKD would be evident and detectable prior to an elevation in plasma creatinine or development of proteinuria is a clinically relevant question, but remains to be determined and is beyond the scope of this study.

In conclusion, 45 min unilateral renal IR without contralateral nephrectomy resulted in rapid upregulation of renal vascular ET‐1 and poor long‐term recovery of renal perfusion, renal atrophy including loss of tubular epithelium, and renal fibrosis. Prior and continuing treatment with an ET_A_ receptor‐selective antagonist or knockout of endothelial ET‐1 was insufficient to prevent the progression of renal injury in this model. These findings highlight the need for research into additional mediators of AKI to CKD progression.

## Conflict of Interest

The author received research funding from Abbvie, Inc. for a separate series of studies investigating the role of endothelin in acute renal failure.
